# Evaluation of the Thermal and Shrinkage Stresses in Restrained High-Performance Concrete

**DOI:** 10.3390/ma12223680

**Published:** 2019-11-08

**Authors:** Yang Yang, Linhao Ma, Jie Huang, Chunping Gu, Zhenjian Xu, Jintao Liu, Tongyuan Ni

**Affiliations:** 1 College of Civil Engineering and Architecture, Zhejiang University of Technology, Hangzhou 310023, China; yangyang@zjut.edu.cn (Y.Y.); 2111706028@zjut.edu.cn (L.M.); 2111706008@zjut.edu.cn (J.H.); jtliu@zjut.edu.cn (J.L.); hznity@zjut.edu.cn (T.N.); 2 Key Laboratory of Civil Engineering Structures & Disaster Prevention and Mitigation Technology of Zhejiang Province, Zhejiang University of Technology, Hangzhou 310023, China; 3 Huzhou Southern Taihu Lake Design Institute of Water Conservancy & Hydro-electric Power Co., Ltd., Huzhou 313000, China; xu_zhenjian@126.com

**Keywords:** high performance concrete, restrained stress, autogenous shrinkage, drying shrinkage, thermal stress

## Abstract

The early age volume deformation is the main course for the cracking of high-performance concrete (HPC). Hence, the shrinkage behavior and the restrained stress development of HPC under different restraints and curing conditions were experimentally studied in this paper. The method to separate the stress components in the total restraint stress was proposed. The total restrained stress was separated into autogenous shrinkage stress, drying shrinkage stress and thermal stress. The results showed that the developments of the free shrinkage (autogenous shrinkage and drying shrinkage) and the restrained stress were accelerated when the drying began; but the age when the drying began did not significantly influence the long-term shrinkage and restrained stress of HPC; the autogenous shrinkage stress continuously contributed to the development of the total restrained stress in HPC; the drying shrinkage stress developed very rapidly soon after the drying began; and the thermal stress was generated when the temperature dropped. The thermal stress was predominant at the early age, but the contributions of the three stresses to the total restrained stress were almost the same at the age of 56 d in this study.

## 1. Introduction

Concrete is prone to cracking due to the early age volume deformation under restrained conditions. Early-age cracking is actually the result of a complex interaction between various phenomena or properties of concrete [1], mainly including the autogenous shrinkage, drying shrinkage, thermal deformation, creep, elastic modulus, tensile strength, etc. These phenomena or properties change rapidly over time due to rapid cement hydration at early ages. The autogenous shrinkage results from the self-desiccation in concrete during the hydration process, and it is more pronounce for concrete with lower water to binder ratio [2,3]. The drying shrinkage is caused by water loss through evaporation. Higher water to binder ratio of concrete would lead to higher drying shrinkage. The temperature of concrete changes at early ages due to heat release during cement hydration and heat exchange with the environment, and the temperature change will result in the thermal deformation of the concrete [2]. Under restraint conditions, the volume deformation (autogenous shrinkage, drying shrinkage and thermal deformation) of concrete will induce stresses (mostly tensile stress) in early age concrete. The tensile stress in concrete is dependent on free volume deformation, elastic modulus, creep, and restraint degree of concrete [2,4]. The stress is proportional to the free volume deformation and elastic modulus of the concrete. The creep is an inherent viscoelastic property of concrete, which could relax the stress and reduce the cracking potential of the concrete structures [5]. The restraint degree would also influence the stress development in concrete. If the concrete is free to deform, stress will not be induced. Higher restrained degree results in higher stress in concrete. When the tensile stress reaches the tensile strength of concrete, the concrete cracks. The early age cracking after the setting of the concrete would reduce the durability and safety of the concrete structures. 

Since the 1990s, high-performance concrete (HPC) has been under continuous development, and has been primarily used in high rise buildings, offshore structures, nuclear power plants and long-span bridges [6,7,8]. However, the early age cracking potential of HPC is much higher than that of normal concrete (NC) [9,10,11,12], because of the lower water to binder ratio and higher content of cementitious materials. The lower water to binder ratio leads to the higher autogenous shrinkage [13,14,15], meanwhile the higher content of cementitious materials results in the higher hydration heat and temperature rise [16]. Numerous studies have paid attention to the early age cracking sensitivity or cracking potential of concrete [10,11,16,17,18,19,20]. The early age shrinkage, heat release and creep of concrete were normally the main concerns of these studies [5,21,22,23]. The evolution of the restrained stress in concrete was also studied, mostly with the ring tests [24,25,26,27,28]. The early age cracking of cement-based materials in the ring tests mainly resulted from the stress caused by the (autogenous and drying) shrinkage. The effects of the expansive agent, light weight aggregate, super absorbent polymers, fly ash, etc. on the residual stress development and cracking behavior of cement-based materials have been investigated with ring tests [29,30,31]. Nevertheless, the ring tests cannot account for the effect of early age temperature variation on the cracking potential of cement-based materials, i.e., the effect of thermal stress was not taken into account [32]. In order to investigate the effect of the thermal stress on the cracking potential of concrete, the temperature stress testing machine (TSTM) was adopted in many studies [19,20,33,34,35,36]. TSTM could evaluate the cracking resistance of mass concrete, in which the temperature drop is the main cause of the cracking. However, TSTM tests were usually conducted under the sealed condition, hence the effect of drying shrinkage on the cracking potential of concrete cannot be revealed with TSTM. Cusson and Hoogeveen also developed an experimental approach to study the cracking behavior of an HPC structure under restraint conditions [37]. The evolution of shrinkage, thermal expansion, elastic modulus and creep of the HPC at early ages could be determined with this approach. Faria et al. invented a variable restraint frame device to investigate the viscoelastic behavior of concrete under restraints to the drying shrinkage deformation. With this test method, the elastic strain, tensile creep and tensile stress induced by restrained shrinkage and elastic modulus of the early age concrete could be assessed simultaneously [4]. 

The cracking of early age concrete was normally caused by the coupled effects of autogenous shrinkage, drying shrinkage and thermal deformation. Wei and Hansen studied the early age strain–stress development in uniaxially restrained cement pastes and concretes with a specially designed testing frame [38]. It was found that the thermal deformation was the main course for the early age stress development in low water to cement ratio cement pastes, and the autogenous shrinkage is the major contributing factor for the tensile stress development in concrete. For HPC, the effect of autogenous shrinkage might be more significant than NC [13,14]. The applications of HPC are increasing, while the respective effect of autogenous shrinkage, drying shrinkage and thermal deformation on the stress development in restrained HPC was rarely studied. 

In this paper, the effect of environmental conditions (sealed and drying) on the volume deformation of HPC was experimentally studied. The restrained stresses aroused by volume deformation of HPC under the rebar restraint and U-steel restraint were measured, and the total restrained stresses were separated into autogenous shrinkage stress, drying shrinkage stress and thermal stress based on the test results. Moreover, the developments of these three stresses and their portions in the total restrained stress were evaluated. 

The results of this study could reveal the main cause of the early age cracking of HPC structures and could provide guidance for the proportion design of HPC from the point of view of cracking potential. Moreover, this study could help to develop cracking control technologies for HPC structures, concerning the structural design, curing, temperature management, etc. Compared with TSTM, the proposed method is easier to perform to determine the proper anti-cracking technologies for concrete structures. 

## 2. Materials and Methods

### 2.1. Materials

**Figure 1:**
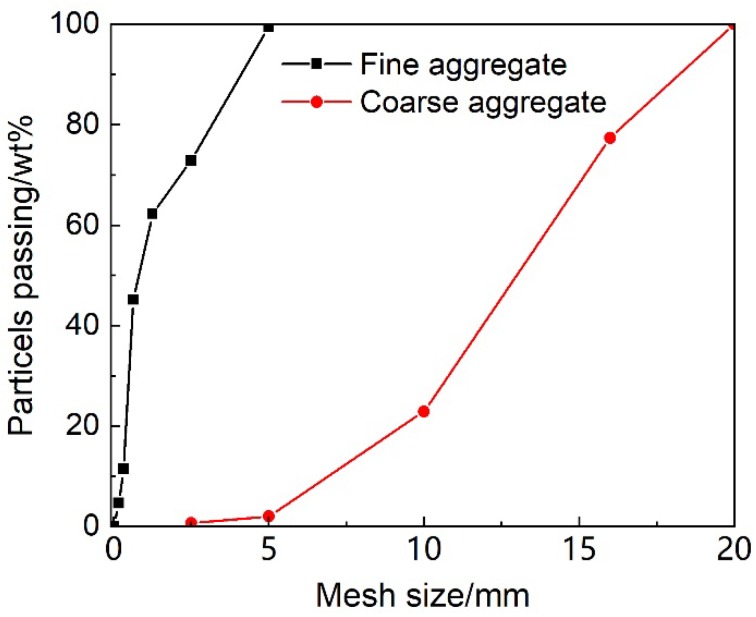
The particle size distribution curves of the aggregates.

The raw materials in this study included P·O 42.5 Portland cement, river sand with a fineness of 3.0, gravels with sizes in the range of 5–20 mm, superplasticizer and water. The chemical composition of the cement is shown in Table 1. The specific gravity of the sand and gravel are 2.60 and 2.70, respectively. The particle size distribution curves of the aggregates are shown in Figure 1. The absorption of the coarse aggregates was 0.85%. The proportion, slump and compressive strength at 28 d of HPC are shown in Table 2.

### 2.2. Environmental Conditions

In this study, two series of experiments, i.e., free shrinkage tests and restrained stress tests, were performed. The environmental temperature and humidity were controlled constantly at 20 ± 2 °C and 60 ± 5% for all the experiments. Four curing regimes were applied on the concrete specimens in the tests, i.e., sealed curing, drying from 1 d (D1), drying from 3 d (D3) and drying from 7 d (D7). Under sealed condition, the specimens were covered with aluminum foil to prevent water evaporation. “Drying from 1 d (D1)” meant that the concrete specimens were sealed before the age of 1 d, and after 1 d, the specimens were exposed to the environment, in which the humidity was 60 ± 5%. As the same as D1, the exposure started from 3 d and 7 d for D3 and D7, respectively.

### 2.3. Free Shrinkage Test

**Figure 2:**
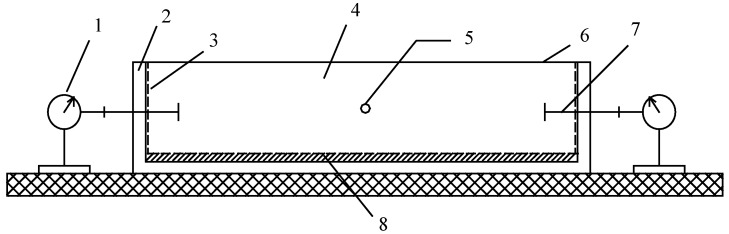
Illustration of the device for free shrinkage test before 1 d: (1) Digital dial gauge; (2) Dismountable mold; (3) Foamed plastic sheet; (4) Concrete specimen; (5) Thermal couple; (6) Plastic film; (7) Gauge studs; (8) 0.5mm-thick Teflon sheet.

In this study, the free shrinkage meant the deformation of concrete specimens without any restraint. It mainly consisted of the autogenous shrinkage and drying shrinkage of the concrete. The free shrinkage tests were performed based on the method proposed by Japan Concrete Institute (JCI) [39]. The size of the specimens was 100 mm × 100 mm × 400 mm, and two specimens were used for the free shrinkage test. Before the age of 1 d, the shrinkage of concrete was measured within the steel mold as shown in Figure 2. Plastic film was used to seal the specimens once they were casted in the steel molds. A thermal couple was embedded in the center of the specimen to record the variation of internal temperature. The thermal deformation was subtracted when calculating the free shrinkage of the specimen. The Teflon sheet was used to reduce the friction between the specimen and the molds. The deformation of the specimens was recorded with digital dial gauges from the age of 12 h, when the stress started to be aroused in restrained concrete according to our trial restrained stress tests. At the age of 1 d, the steel mold was removed, and the shrinkage of the specimens was recorded with digital dial gauges until the age of 56 d. For sealed curing, the concrete specimens were sealed all the time during the tests. Our previous study showed that, after sealing for 65 days, the weight loss of the specimens was between 0.034–0.075% [5], which indicated that the sealing method was very effective. This method has been successfully used for the measurement of the autogenous shrinkage of concrete [13]. For D1, D3 and D7 conditions, the specimens were exposed to the environment from the age of 1 d, 3 d and 7 d. 

### 2.4. Retrained Stress Test

**Figure 3:**
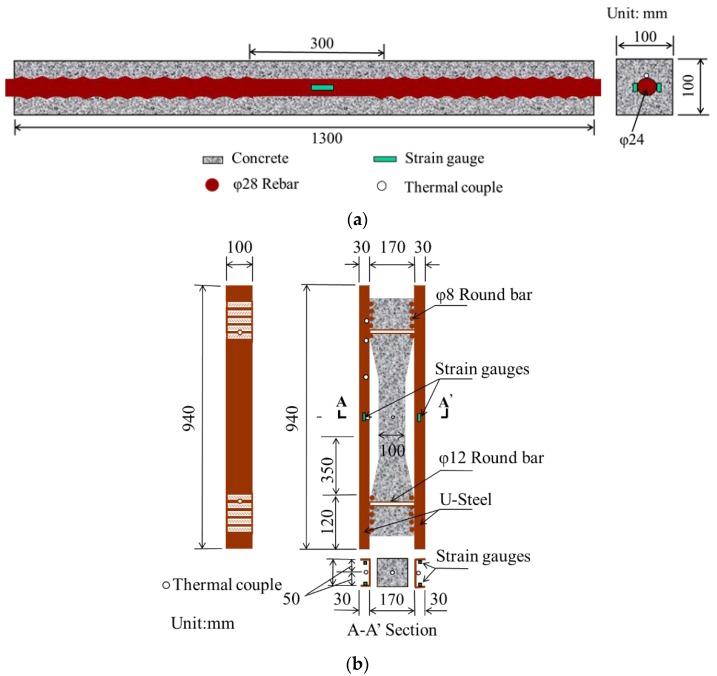
Illustrations of rebar restraint and U-steel restraint: (**a**) Rebar restraint; (**b**) U-steel restraint.

In order to separate the restrained stress caused by different types of volume deformation, two types of restraint (i.e., rebar restraint and U-steel restraint) were adopted in the restrained stress tests. The rebar restrained stress tests were performed based on the method proposed by JCI [39], and the U-steel restrained stress tests were conducted according to JISA 1151-2011 [40]. Details of the tests are shown in Figure 3, and two specimens were used for both the rebar and U-steel restrained stress tests. Similar methods have been applied to study the cracking potential of ultra-high-performance concrete under different conditions [41,42].

In the rebar restrained stress tests (as shown in Figure 3a), the size of the specimen was 100 mm × 100 mm × 1300 mm and the concrete was restrained with a rebar whose diameter was 28 mm. The middle of rebar was polished in order to adhere the strain gauges on the rebar, so that the strain of the rebar during the tests could be recorded. The restrained stress in concrete could be calculated with Equation (1).

(1) $$\sigma_{C}=\frac{E_{s}\varepsilon_{s}A_{s}}{A_{c}},$$

where, *σ_c_* means the restrained stress in concrete (MPa); *E_s_* is the elastic modulus of the rebar, which was 2.10 × 10^5^ MPa; *ε_s_* is the strain of the rebar, which was recorded by the strain gauges; *A_s_* is the section area of the rebar (mm^2^); and *A_c_* is the section area of the concrete specimen (mm^2^). A thermal couple was placed around the rebar to detect the temperature of the rebar and concrete. 

The test method for U-steel restraint is shown in Figure 3b. The restraint was exerted by the round bars at the two ends of the U-steel. Strain gauges were adhered on the U-steel to measure the strain of the U-steel. The restrained stress in concrete also can be calculated with Equation (1) once *E_s_*, *ε_s_* and *A_s_* were replaced with the elastic modulus, strain and section area of the U-steel. Thermal couples were used to record the temperature variations of the concrete and U-steel. 

The trial tests showed that the restrained stress was aroused at the age of about 12 h, hence the strain and the temperature data were recorded from 12 h and till 56 d with a data logger. The molds for the restrained stress tests were also removed at the age of 1 d, as the same as that in the free shrinkage tests. Trial tests showed that the removal of the molds did not influence the strain data of the rebar and the U-steel. The sealed, D1, D3 and D7 conditions were also applied on the specimens in both two types of restrained stress tests. 

The main concern of this study was the restrained stress in the central segment of the rebar and U-steel restrained concrete specimens. The size of this segment was both 100 mm × 100 mm × 300 mm for the rebar and U-steel restrained tests. The reinforcement ratios for these two methods were almost the same, i.e., 4.74% for rebar restraint and 4.80% for U-steel restraint. The difference between these two methods lies in the existence of the thermal stress. The concrete and the steel have similar thermal expansion coefficients, which are both about 10–12 με/K [43]. Hence, the thermal stress was not aroused in the rebar restrained stress tests since the temperatures of the concrete and the rebar were almost the same in the tests. The stress in the rebar was mainly caused by the (autogenous and drying) shrinkage of the concrete. In the U-steel restrained stress tests, the central segment of the concrete specimen and the U-steel were not contacted, so temperature difference was existed between the U-steel and concrete. Consequently, besides the shrinkage stresses, the thermal stress was also aroused in the U-steel restrained concrete specimens.

## 3. Results

### 3.1. Free Shrinkage of HPC

**Figure 4:**
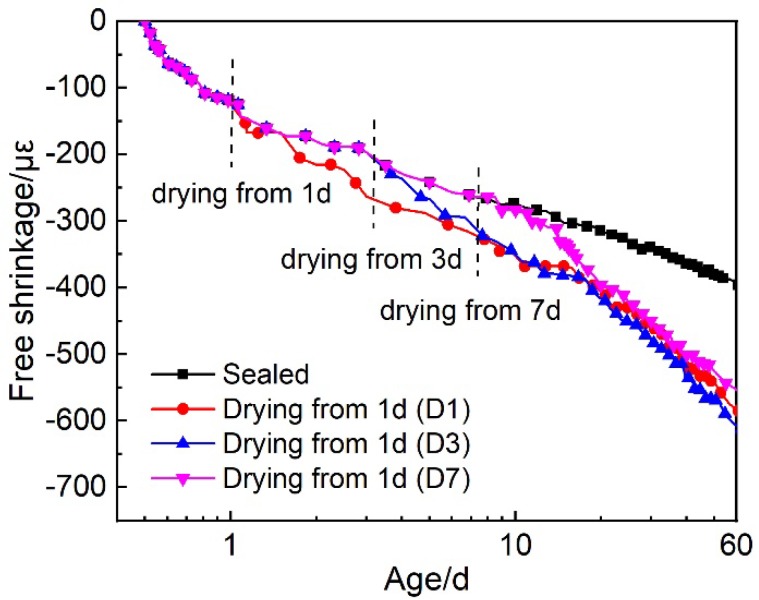
Free shrinkage of HPC under various curing conditions.

The free shrinkage of HPC under different curing conditions are shown in Figure 4. Under sealed condition, the deformation of HPC was resulted from the autogenous shrinkage. It can be seen that the autogenous shrinkage developed quite fast at early ages, especially before the age of 1 d. The autogenous shrinkage of HPC at 1 d was about 120 με, which is 35% of that at 28d. The hydration of cement proceeded very fast at the very early ages, as well as the self-desiccation and the decrease of the internal humidity. Hence, the autogenous increased very rapidly at early ages.

Under the conditions of D1, D3 and D7, the shrinkage after drying can be assumed to be the sum of the autogenous shrinkage and the drying shrinkage. It can be seen from Figure 4 that the drying shrinkage was also quite remarkable, although HPC has a low water to cement ratio and water usage. Due to the rapid water release, the drying shrinkage increased relatively fast after the beginning of the drying. In addition, it is interesting that, at late age, i.e., after 20d, no matter when the drying began, the total shrinkage of HPC was almost the same. The age when the drying began, showed influences on the shrinkage development soon after the drying, while it did not affect the long-term shrinkage of HPC. The development of drying shrinkage is related to the pore structure and the internal humidity of concrete. If the drying of concrete started at a later age, the internal humidity of concrete would be lower and the pores in concrete would be finer, then the water release would result in more remarkable drying shrinkage. At the late ages, the HPCs under different drying conditions may had similar pore structure and internal humidity, hence HPCs under drying conditions showed almost the same free shrinkage.

### 3.2. Restrained Stresses in HPC

#### 3.2.1. Restrained Stresses under Sealed Condition

**Figure 5:**
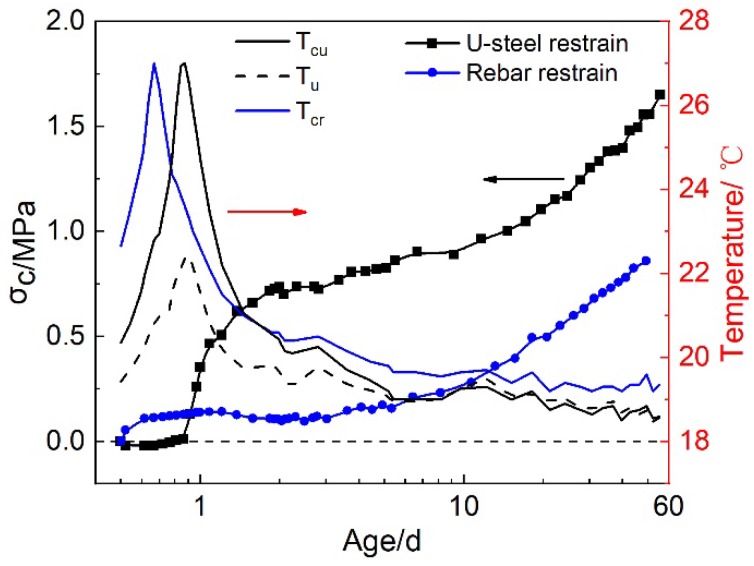
The restrained stresses in HPC under different restraints and the temperature variations in restrained HPC specimens and the U-steel (T_cr_: the average temperature of HPC specimens and the rebar in rebar restrained stress test; T_cu_: the average temperature of HPC specimens in U-steel restrained stress test; T_u_: the average temperature of U-steel in U-steel restrained stress test).

Based on the strain data of the rebar or U-Steel, the restrained stress in HPC could be calculated with Equation (1). Under sealed condition, the restrained stress was mainly aroused by the autogenous shrinkage and the thermal deformation. The comparison of the restrained stresses in HPC under rebar and U-steel restraint, as well as the temperature variations in restrained HPC specimens and the U-steel, are shown in Figure 5. It can be seen that the restrained stress in HPC specimens under U-steel restraint was higher than that under rebar restraint, although the reinforcement ratios were almost the same for two restraint conditions. This was resulted from the thermal stress that aroused in HPC specimen under U-steel restraint. The temperature of HPC specimen under U-steel restraint rose by about 7 °C due to hydration heat release before the age of about 1 d, while the temperature of U-steel only rose by about 4 °C. Hence the temperature difference resulted in the compressive restrained stress in HPC specimen before the age of 1 d. Because the room temperature was controlled at 20 ± 2 °C, the temperature of HPC specimen began to drop after the age of 1 d. The temperature drop of HPC specimen was also higher than that of U-steel, hence tensile restrained stress was aroused and increased very fast in U-steel restraint specimen during the temperature dropping process. The temperatures of HPC specimen and rebar were almost the same in the rebar restraint stress test, so quick development of the restraint stress due to the temperature drop was not observed in HPC specimens under rebar restraint. The development of the restraint stress in HPC specimen under rebar restraint was resulted from the autogenous shrinkage of HPC. So, it was increasing continuously, and had a similar trend to the development of the autogenous shrinkage of HPC.

#### 3.2.2. Restrained Stresses under Drying Conditions (D1, D3 and D7)

**Figure 6:**
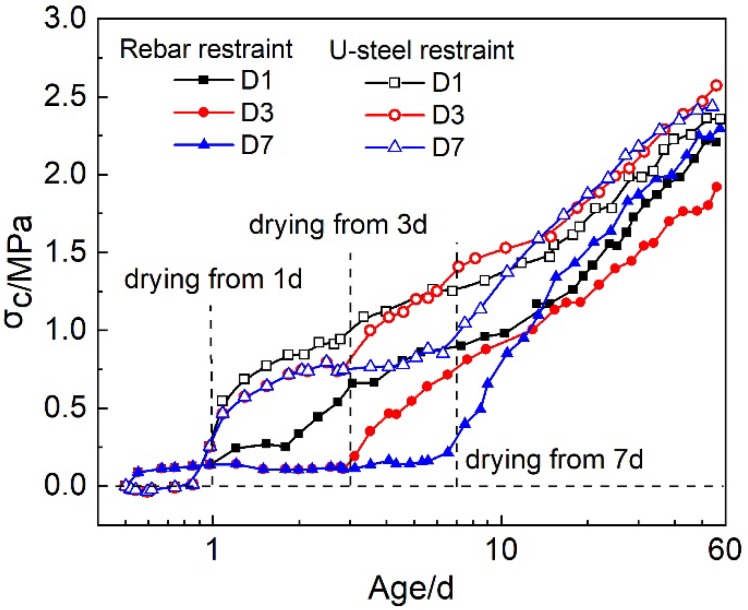
The restrained stresses in rebar and U-steel restrained HPC specimens under drying condition.

Under the drying conditions, the drying shrinkage would also contribute to the development of the restrained stress. Figure 6 shows the restrained stresses in rebar and U-steel restrained HPC specimens under drying conditions.

Under the rebar restraint, the restrained stress before the drying was the same to that under sealed condition. When the drying began, the restrained stress notably improved. At the age of 56 d, the restrained stress in rebar restrained HPC specimen did not show much difference, which implied that the age when the drying began did not show significant influence on the long term restrained stress in the HPC. Nevertheless, the development speed of the restrained stress soon after the drying began was not the same for conditions D1, D3 and D7. It is shown that the development speed of the restrained stress under rebar restraint followed D7>D3>D1. This may result from the increasing elastic modulus of HPC at early ages. The same strain might lead to higher stress in HPC with higher elastic modulus. In general, the age when the drying began influenced the development speed of the restrained stress in HPC at early ages. The earlier the drying began, the lower the development speed of the restrained stress at early ages was. 

Under the U-steel restraint, the effect of the age when the drying began on the restrained stresses in U-steel restrained HPC specimen was similar to that in rebar restrained specimens. To begin the drying earlier led to the lower development speed of the restrained stress at early ages, and the age when the drying began did not show much influence on the restrained stress at late age. Due to the presence of thermal stress, the restrained stresses in U-steel restrained HPC specimens at 56 d were higher than those in rebar restrained specimens.

### 3.3. Separation of the Shrinkage and Thermal Retrained Stresses

In order to evaluate the influence of the shrinkage and the thermal deformation on the restrained stress in HPC, the restrained stresses aroused by autogenous shrinkage, drying shrinkage and thermal deformation were separated based on the test results of the restrained stress tests.

#### 3.3.1. The Method of Separation

In order to separate the restrained stress, the following assumptions were made: 

(1) The degree of rebar and U-steel restraints were considered to be the same;

(2) The autogenous shrinkage stress equaled to the rebar restrained stress under sealed condition;

(3) The temperature variations of HPC specimens under sealed condition and drying conditions were the same;

(4) The autogenous shrinkage was not influenced by the drying, i.e., the autogenous shrinkage of HPC under sealed and drying conditions was considered to be the same.

Based on these assumptions, the restrained stresses could be separated according to the equations presented in Table 3. 

#### 3.3.2. The Separation of Autogenous Shrinkage and Drying Shrinkage Stresses Based on Rebar Restrained Stress Test under Drying Condition

**Figure 7:**
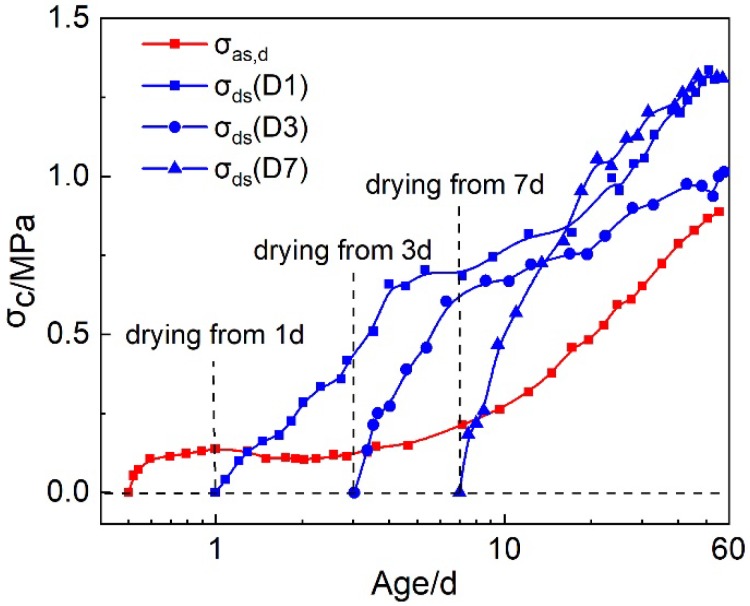
The development of the autogenous shrinkage stress and drying shrinkage stress under rebar restraint and different drying conditions.

Under the drying condition, the rebar restrained stress was composed of the autogenous shrinkage stress and the drying shrinkage stress. Based on the method proposed in Table 3, the autogenous shrinkage stress and the drying shrinkage stress were calculated. The developments of the autogenous shrinkage stress and the drying shrinkage stress were shown in Figure 7. The autogenous shrinkage stress was assumed to be equaled to the restrained stress of HPC specimens under rebar restraint and sealed condition, and it was not influenced by the drying. The drying shrinkage developed very fast after the beginning of the drying and continued to increase at late ages. In order to evaluate the influences of the autogenous and drying shrinkage on the restrained stress in HPC, the proportions of the autogenous and drying shrinkage stress in the total restrained stress were calculated, which is shown in Table 4. It can be seen that the drying shrinkage stress reached 70–80% of the total restrained stress very rapidly after the drying began, i.e., at early ages, the drying shrinkage were main course for the cracking of HPC. Hence, careful curing is quite important for reducing the cracking potential of HPC at early ages. At late ages, the proportions of drying shrinkage stress and autogenous shrinkage stress were 50–60% and 40–50%, respectively. The autogenous shrinkage stress increased a little faster than the drying shrinkage stress at late ages. Generally, both the drying shrinkage and the autogenous shrinkage were the main causes for the restrained stress in HPC at late ages.

#### 3.3.3. The Separation of Autogenous Shrinkage and Thermal Stresses Based on U-Steel Restrained Stress Test under Sealed Condition

**Figure 8:**
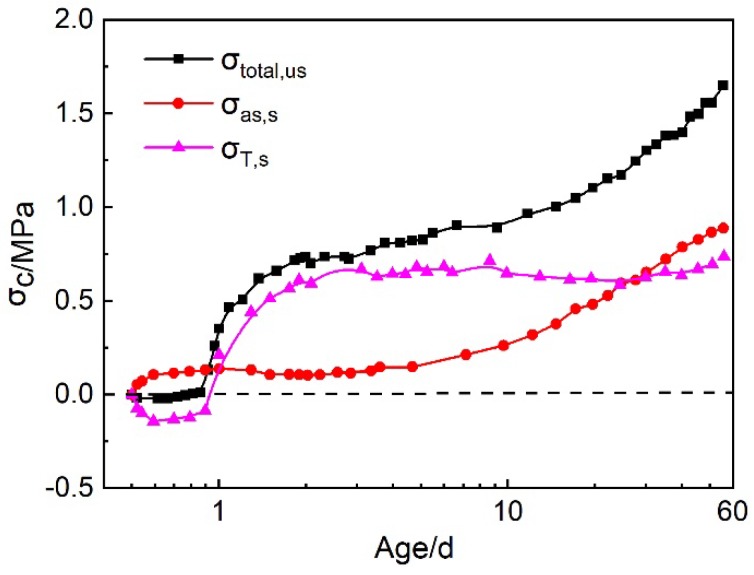
The development of the autogenous shrinkage stress, thermal stress and the total restrained stress under U-streel restraint and sealed condition.

Under the sealed condition, the U-steel restrained stress consisted of the autogenous shrinkage stress and the thermal stress. The developments of these two stresses and the total restrained stress were shown in Figure 8. It can be seen that the thermal stress developed with the temperature variation. Temperature rise led to the compressive restrained stress, and temperature drop resulted in the tensile restrained stress. At early ages, after the temperature of concrete began to drop, the tensile thermal stress became more prominent than the autogenous shrinkage stress. At late ages, the thermal stress stopped increasing due to the stable temperature in HPC. The proportions of autogenous shrinkage stress and thermal stress in total restrained stress are shown in Table 5. The proportion of the thermal stress improved quickly to 60–85% of the total restrained stress after the thermal stress turned into tensile stress from compressive stress. So, under the presented condition, the thermal stress was the predominant cause for the cracking of HPC at early ages. In a real HPC structure, the temperature variation will be more remarkable, hence the influence of the thermal stress will be more significant. At late ages, the autogenous shrinkage stress was still arising, hence its proportion grew to 54% at the age of 56 d, which was even higher than the thermal stress.

#### 3.3.4. The Separation of Autogenous Shrinkage, Drying Shrinkage and Thermal Stresses Based on U-Steel Restrained Stress Test under Drying Condition

**Figure 9:**
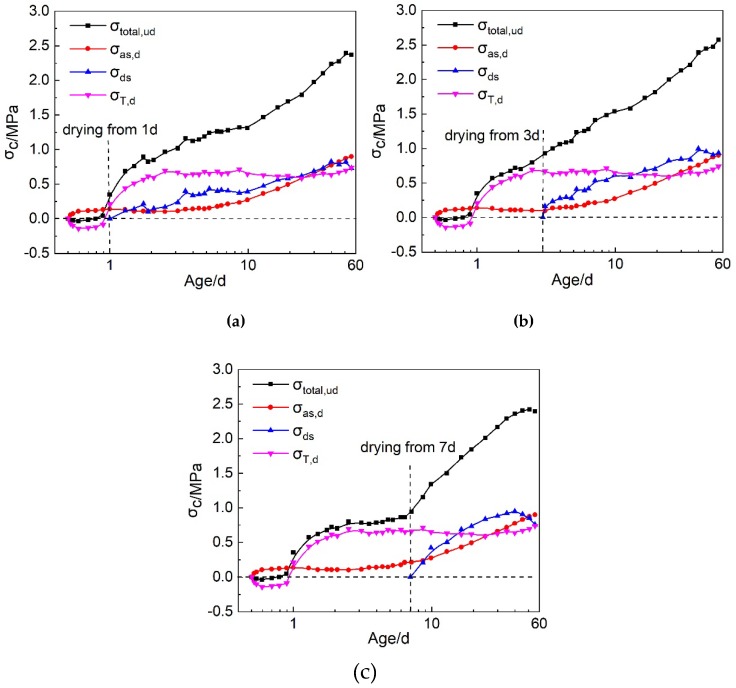
The development of the autogenous shrinkage stress, drying shrinkage stress, thermal stress and the total restrained stress under U-streel restraint and different drying conditions: (**a**) Drying from 1 d; (**b**) Drying from 3 d; (**c**) Drying from 7 d.

Under the drying condition, the U-steel restrained stress can be divided into autogenous shrinkage stress, drying shrinkage stress and thermal stress. The comparison of three types of stresses and the total restrained stress were shown in Figure 9. At early ages, after the age of 1 d, the thermal stress that originated from the temperature drop of HPC was more predominant, comparing with the autogenous shrinkage and drying shrinkage stresses. The drying shrinkage also developed very fast and became higher than the autogenous shrinkage stress soon after the drying begin. At the age of 56 d, the autogenous shrinkage stress, drying shrinkage stress and thermal stress were close to the others in this study. Autogenous shrinkage, drying shrinkage and thermal deformation all contributed to the restrained stress in HPC at late ages. Table 6 shows the proportions of autogenous shrinkage stress, drying shrinkage stress and thermal stress in total restrained stress. It can be seen that at early ages, soon after the thermal stress changed into tensile stress, its proportion improved very fast and reached 60–90% of the total restrained stress. The thermal stress was also the main cause for the early age cracking of HPC under this circumstance. After the drying began, the proportion of the drying shrinkage stress rose to about 40% of the total restrained stress, which would also contribute to the cracking of HPC. At the age of 56 d, the autogenous shrinkage stress, drying shrinkage stress and thermal stress were all 30–40% of the total restrained stress. They were all important for the restrained stress in HPC at late ages.

### 3.4. The Roles of the Autogenous Shrinkage, Drying Shrinkage and Thermal Deformation on the Restrained Stress in HPC

Due to the lower water to binder ratio and higher content of cementitious materials, HPC normally exhibits higher autogenous shrinkage than that of NC. Consequently, the effect of autogenous shrinkage on the restrained stress development in HPC will be more significant than that in NC. The results of the restrained stress tests in this study showed that the autogenous shrinkage resulted in 10–30% of the total restrained stress under different conditions at early ages. Moreover, the autogenous shrinkage stress increased continuously during the tests, and was as high as drying shrinkage stress and thermal stress at late ages. So, in order to eliminate the cracking risk of HPC, the autogenous shrinkage has to be considered when designing HPC. 

The drying shrinkage significantly influences the restrained stress in HPC, especially soon after the drying begins. In this study, the drying shrinkage contributed more than 30% of the total restrained stress at early ages under different drying conditions. Hence, curing HPC under sealed or moisture conditions as long as possible could help to improve the cracking resistance of HPC. Longer curing could make HPC strong enough to resist the restrained stress development when the drying begins. 

The thermal stress aroused by the temperature drop is an important part of the total restrained stress in HPC. In this study, the thermal stress was predominant at early ages, and was close to drying and autogenous shrinkage stresses at late ages. Hence, in order to reduce the cracking potential of HPC, it is essential to reduce the thermal stress in HPC structures, particularly at early ages. Generally, the methods to reduce the cracking risk caused the thermal stress are to lower or to postpone the temperature drop, which would reduce the thermal stress that causing the cracking.

## 4. Conclusions

The following conclusions were drawn from the obtained results.

1. The method to separate the stress components in the total restrained stress was proposed. With this method, the restrained stress was separated into autogenous shrinkage stress, drying shrinkage stress and thermal stress. 

2. Under sealed condition, the restrained stress in HPC specimens under U-steel restraint was higher than that under rebar restraint, due to the existence of thermal stress. The autogenous shrinkages stress and the thermal stress could be separated from the total restrained stress. The thermal stress contributed 60–85% of the total tensile stress at the early ages (before the age of 7 d), and at the late age (56 d), the thermal stress and autogenous shrinkage stress contribute 46% and 54% to the total restrained tensile stress respectively in HPC.

3. Under drying conditions, the age when the drying began influenced the development speed of the restrained stress in HPC at early ages, but it did not show much influence on the restrained stress at late ages. The thermal stress dominates the restrained tensile stress development in HPC at the early ages. It was as high as 60–88% of the total restrained stress before the age of 7 d. After the drying began, the portion of the drying shrinkage stress rose to about 40% of the total restrained stress very rapidly. At the age of 56 d, the autogenous shrinkage stress, drying shrinkage stress and thermal stress all contributed 30–40% to the total restrained tensile stress. 

## Figures and Tables

**Table 1:** Chemical composition of the cement (wt%).

CaO	SiO_2_	Al_2_O_3_	Fe_2_O_3_	MgO	SO_3_	K_2_O	Na_2_O	TiO_2_	Loss
55.10	21.40	6.30	4.30	2.80	2.41	0.46	0.23	0.42	2.72

**Table 2:** Mix proportion, slump and 28d compressive strength of HPC.

Water to Cement Ratio	Mix Proportion/kg/m^3^					Slump /mm	28d Compressive Strength/MPa
	Cement	Water	Fine Aggregate	Coarse Aggregate	Superplasticizer		
0.28	550	154	644	1052	12.1	185	84.4

**Table 3:** Separation of the restraint stresses.

Restraint Method	Stress Components and Abbreviations	Calculation of the Stress Components	
		Sealed Condition	Drying Condition
**Rebar restraint**	Total stress *σ_total_*	*σ_total,rs_*	*σ_total,rd_*
	Autogenous shrinkage stress *σ_as_*	*σ_as,s_* = *σ_total,rs_*	*σ_as,d_* = *σ_as,s_*
	Drying shrinkage stress *σ_ds_*	---	*σ_ds_* = *σ_total,rd_* − *σ_as,d_*
	Thermal stress *σ_T_*	---	---
**U-steel restraint**	Total stress *σ_total_*	*σ_total,us_*	*σ_total,ud_*
	Autogenous shrinkage stress *σ_as_*	*σ_as,s_* = *σ_total,rs_*	*σ_as,d_* = *σ_as,s_*
	Drying shrinkage stress *σ_ds_*	---	*σ_ds_* = *σ_total,ud_* − *σ_as,d_* − *σ_T,d_*
	Thermal stress *σ_T_*	*σ_T,s_* = *σ_total,us_ − σ_as,s_*	*σ_T,d_* = *σ_T,s_*

**Table 4:** The proportions of drying shrinkage stress and autogenous shrinkage stress in total restrained stress under rebar restraint and different drying conditions.

The Age When the Drying Began	Type of the Restrained Stresses	Age					
		1 d	3 d	7 d	14 d	28 d	56 d
1 d	Autogenous shrinkage stress	100%	20%	23%	31%	36%	40%
	Drying shrinkage stress	0%	80%	77%	69%	64%	60%
3 d	Autogenous shrinkage stress	100%	100%	26%	33%	43%	48%
	Drying shrinkage stress	0%	0%	74%	67%	57%	52%
7 d	Autogenous shrinkage stress	100%	100%	100%	30%	34%	41%
	Drying shrinkage stress	0%	0%	0%	70%	66%	59%

**Table 5:** The proportions of autogenous shrinkage stress and thermal stress in total restrained stress under U-streel restraint and sealed condition.

Type of the Restrained Stresses	Age					
	1 d	3 d	7 d	14 d	28 d	56 d
Autogenous shrinkage stress	40%	16%	23%	36%	50%	54%
Thermal stress	60%	84%	77%	64%	50%	46%

**Table 6:** The proportions of autogenous shrinkage stress, drying shrinkage stress and thermal stress in total restrained stress under U-streel restraint and different drying conditions.

The Age When the Drying Began	Type of the Restrained Stresses	Age					
		1 d	3 d	7 d	14 d	28 d	56 d
1 d	Autogenous shrinkage stress	40%	11%	16%	26%	33%	38%
	Drying shrinkage stress	0%	22%	32%	33%	34%	31%
	Thermal stress	60%	67%	52%	41%	32%	31%
3 d	Autogenous shrinkage stress	40%	12%	15%	24%	30%	34%
	Drying shrinkage stress	0%	0%	42%	41%	41%	39%
	Thermal stress	60%	88%	43%	35%	29%	27%
7 d	Autogenous shrinkage stress	40%	12%	23%	26%	30%	38%
	Drying shrinkage stress	0%	0%	0%	35%	41%	32%
	Thermal stress	60%	88%	77%	39%	29%	30%
